# Is organizational progress in the EFQM model related to employee satisfaction?

**DOI:** 10.1186/1472-6963-14-468

**Published:** 2014-10-24

**Authors:** Carmen Matthies-Baraibar, Andoni Arcelay-Salazar, David Cantero-González, Alberto Colina-Alonso, Marbella García-Urbaneja, Rosa María González-Llinares, Jon Letona-Aranburu, Catalina Martínez-Carazo, Maider Mateos-del Pino, Roberto Nuño-Solinís, Esther Olaetxea-Urizar, José Antonio de la Rica-Giménez, María Angeles Rodríguez-González, Silvia Dabouza-Acebal

**Affiliations:** Basque Health Service (Osakidetza), Vitoria-Gasteiz, Spain; Basque Institute for Healthcare Innovation (O+berri), Torre del BEC, Ronda de Azkue 1, 48902 Barakaldo, Spain

**Keywords:** EFQM model, Total quality, Professional satisfaction, Quality improvement, Excellence

## Abstract

**Background:**

To determine whether there is greater employee satisfaction in organisations that have made more progress in implementation of the European Foundation for Quality Management (EFQM) model.

**Methods:**

A series of cross-sectional studies (one for each assessment cycle) comparing staff satisfaction survey results between groups of healthcare organisations by degree of implementation of the EFQM model (assessed in terms of external recognition of management quality in each organisation). Setting: 30 healthcare organisations including hospitals, primary care and mental health providers in Osakidetza, the Basque public health service. Participants: Employees of 30 Osakidetza organisations. Intervention: Progress in implementation of EFQM model. Main outcome measures: Scores in 9 dimensions of employee satisfaction from questionnaires administered in healthcare organisations in 4 assessment cycles between 2001 and 2010.

**Results:**

Comparing satisfaction results in organisations granted Gold or Silver Q Awards and those without this type of external recognition, we found statistically significant differences in the dimensions of training and internal communication. Then, comparing recipients of Gold Q Awards with those with no Q Certification, differences in leadership style and in policy and strategy also emerged as significant.

**Conclusions:**

Progress of healthcare organisations in the implementation of the EFQM Excellence Model is associated with increases in their employee satisfaction in dimensions that can be managed at the level of each organisation, while dimensions in which no statistically significant differences were found represent common organisational elements with little scope for self-management.

## Background

The public Basque Health Service, Osakidetza, is a public body run by the Regional Government of the Basque Country with the mission of providing health services to the population of the autonomous region, around 2,200,000 people. Osakidetza is formed by a set of service provider organisations (including acute hospitals, medium- and long-stay hospitals, psychiatric hospitals, primary care organisations, mental health networks, and integrated healthcare organisations, as well as a centre for transfusion and human tissues and an emergency services provider), the number and structure of which have varied over the course of the study (from 31 service providers in 2001 to 26 in the present day).

The healthcare organisations that compose the Basque Health Service are granted different levels of independence and responsibility depending on the management area. Hence, while some areas, such as strategic policies, and the contracting and payment of staff, are coordinated/centralised at the corporate level; responsibility for others, such as the development of local management plans, training, the organisation of processes and the management of alliances, is decentralised, with the separate healthcare organisations being allowed a greater level of independence.

Osakidetza has been involved in quality management activities since 1992. Some of the first milestones along this path were becoming a partner of Euskalit, the Basque Foundation for Quality Promotion [1] (an organisation that promotes improvements in management in the Basque region) and adopting in 1995 the European Foundation Quality Management (EFQM) Excellence Model [2] as a model of reference for total quality.

At the corporate level, the adoption of the EFQM model was driven by a range of strategies and measures, including the adaptation of the model to the healthcare setting, an extensive programme of staff training in this management model and the establishment of a schedule of two-yearly self-assessments, through which healthcare organisations are encouraged to use the EFQM model on a voluntary basis [3–6]. At the same time, a series of tools and methodologies were adapted and introduced to favour the rolling-out of the EFQM model [7], including the 5-s principles [8], management by processes [9–11], the development of strategic plans [12–14], satisfaction surveys with both patients and health service staff [15], balanced scorecards, and ISO 9000, 14000 and 18000 certification, as well as other activities in the fields of management innovation and social corporate responsibility.

In this general corporative context, the EFQM has not been adopted evenly across the health service, with healthcare organisations progressing at different paces and reaching different degrees of implementation of the model. In line with this, a range of external awards have been received by different organisations, including recognitions both regionally (7 Gold and 21 Silver Qs for Quality from the Basque Government) and internationally (1 European Special Prize and 2 Iberoamerican Quality Prize).

One of the areas that the EFQM model aspires to influence is the perception by staff of the organisation in which they work (EFQM criterion 7 a) [2]. Given this, 15 years after Osakidetza decided to adopt the Total Quality Principles and EFQM Excellence Model to drive improvements, this study set out to explore whether there was a relation between the degree of implementation of the model in different healthcare organisations of the regional service and the mean level of satisfaction among employees in these organisations.

There is evidence in the literature of a positive association between use of the EFQM model and the performance of organisations in the industrial sector [16–18], with studies generally focussing on economic and financial results [16, 18]. In the health sector, however, as revealed by the review of Minkman et al. [19], there is only weak evidence of the impact of this model, it being almost exclusively limited to descriptive accounts. These include descriptive studies of the progress in rolling out the EFQM model in healthcare organisations in Osakidetza [4, 20].

There is some more sound evidence from the USA, regarding the Baldrige Health Care Criteria Framework, a total quality management similar to the EFQM model. Specifically, a study [21] in 220 hospitals demonstrated a significant positive association between meeting the criteria in the various categories of the framework and performance in the results’ dimensions. In particular, the strongest association was found between degree of adherence to the quality improvement model and the “staff and work systems results” dimension, this being interpreted by the authors as evidence of a relationship between a quality culture in an organisation and greater employee involvement and satisfaction, consistent also with the results of authors such as Shortell et al. on the positive association between quality improvement implementation and perceived human resource development [22].

In this context, the present study aims to assess whether there is an association between the degree of implementation of the EFQM model in 30 healthcare organisations of Osakidetza, at various time points (on the basis of a series of assessments between 2001 and 2010), and the perception of the health service employees, in terms of their level of satisfaction.

## Methods

A cross-sectional study design was used for each period under consideration. As in previous studies [16, 18], whether an organisation had received (or not) an external quality award was used as a proxy for the degree of implementation of the EFQM model; the granting of an award being regarded as a milestone in the organisation, the culmination of previous efforts to deploy quality management based on this excellence model. Specifically, the external assessments considered were the Silver (>400 points) and Gold (>500 points) Q Awards granted by the Basque Government.

Employee perceptions (EFQM criterion 7a, “People results”) were compared, on the one hand, in the organisations which had obtained some type of Q award (Silver or Gold) and those which had not and, on the other, in organisations given the Gold Q Award (the highest level of excellence in terms of the model considered) and those without any Q recognition.

Assessment of employee perceptions of the organisations in which they work was based on the staff satisfaction surveys that have been carried out periodically by service providers in Osakidetza since 2001. There were various cycles of assessment (2001–2003, 2004–2006, 2007–2008, and 2009–2010), the questionnaire and data collection procedure being modified/adapted in each cycle and, hence, the results are not fully comparable between these periods. For this reason, the statistical analysis was limited to cross-sectional comparisons within each assessment cycle, longitudinal analysis being avoided. For the purposes of this study, the results were standardised across the surveys on a scale of 0 to 10.

The construct validity and reliability of the satisfaction questionnaire used with the health service employees was tested on the basis of a sample of 7,145 questionnaires (corresponding to 28.7% of the entire staff of Osakidetza) conducted in year 2010. On the one hand, principal component analysis was performed confirming the existence of 9 separate dimensions within the satisfaction construct, which together explained 70% of the variance of the construct. Comparisons were made between the organisations for each of these dimensions. Further, the high reliability of the tool was confirmed by calculating Cronbach’s alpha, both for the complete set of items on the questionnaire (0.97) and for the items in each dimension. A description of the items of the questionnaire and the corresponding values of Cronbach’s alpha are listed by dimension in Table 1.

**Table 1:** **Dimensions and items on the staff satisfaction questionnaire used in the Basque health service (2010) and Cronbach’s alpha by dimension from 2010 survey results**

Items by dimension on the staff satisfaction questionnaire used in the Basque health service	Cronbach’s alpha
**Dimension 1. Conditions of health and safety at work**	0.83
(P1).- The physical and environmental characteristics of the workspace (space, temperature, level of noise, lighting, equipment, etc.) are adequate.	
(P2).- There is an adequate standard of safety at work .	
(P3).- The distribution and sharing of the workload is adequate, as is the time available to complete the work.	
(P4).- Conditions are suitable to allow you to concentrate on the task in hand without interruptions.	
(P5).- The level of physical effort required in your daily work is reasonable given the position you hold.	
(P6).- The level of mental or psychological effort required in your daily work is reasonable given position you hold.	
**Dimension 2. Working conditions**	0.84
(P7).- You are satisfied with the conditions in terms of working hours and working days.	
(P8).- The conditions regarding staff leave are reasonable.	
(P9).- The conditions regarding flexibility in working hours, etc. allow you to achieve a good work-life balance.	
(P10).- You are satisfied with the employment stability in your job with Osakidetza.	
(P11).- You feel that the working conditions (working day, leave etc.) are better in Osakidetza than in the private sector.	
(P12).- The mechanisms in Osakidetza for promotion and professional development such as temporary internal promotion, opportunities to practice roles corresponding to more senior positions, terms of service, etc. are adequate.	
**Dimension 3. Training and professional development**	0.89
(P13).- The training and preparation provided in Osakidetza are adequate for the position you hold.	
(P14).- You are satisfied with the training and learning opportunities, for promotion and professional development, offered by your organisation.	
(P15).- You are satisfied by the tasks you perform in your job.	
(P16).- In performing your job, you are able to adequately develop your knowledge and skills.	
(P17).- In your organisation, for those with the same skills and abilities, there are equal opportunities for promotion and professional development.	
(P18).- You believe that in Osakidetza your professional expectations are going to be met.	
(P19).- You feel that the factors considered for the assignment of the level of professional development (work performance and quality, involvement with the organisation, organisational achievements, and personal merits) and the way of evaluating them are adequate.	
**Dimension 4. Pay**	0.88
(P20).- Your total pay is reasonable for the work you do.	
(P21).- Your pay is reasonable compared to other people with an equivalent level of responsibility in other organisations (outside Osakidetza).	
(P22).- Your pay is reasonable compared colleagues from other professional groups in Osakidetza.	
**Dimension 5. Technical and material resources**	0.89
(P23).- The intranet of your organisation is a useful source of information and tool for communication.	
(P24).- The information systems used in you workplace, such as OSABIDE, ALDABIDE, GIZABIDE, ZAINERI and other computer applications, are useful tools.	
(P25).- Your organisation makes good use of new technologies for exchanging experiences, disseminating information and professional guidelines, etc.	
(P26).- You have sufficient and appropriate resources (computers, other technical equipment, consumable materials, etc.) for carrying out your daily work.	
**Dimension 6. Working environment**	0.84
(P27).- In your unit (or service, ward, department, primary care unit, etc.), there is a good working environment in terms of working relationships.	
(P28).- In general, there are good relationships between colleagues working together in your unit.	
(P29).- In general, the working relationships with people from other units are based on collaboration.	
**Dimension 7. Internal communication**	0.90
(P30).- The information you are provided with and the instructions you are given for carrying out your work are sufficient.	
(P31).- You receive sufficient appropriate information regarding any decisions, projects and activities of the organisation that may affect you.	
(P32).- You know who to turn to if you have any concerns regarding your work.	
(P33).- There is a good communication between your line manager and your team regarding projects, problems, and any issues affecting you.	
(P34).- There are sufficient channels for suggestions or complaints to senior management.	
(P35).- You are adequately informed of matters arising in other units related to your work.	
**Dimension 8. Leadership, management and organisation style**	0.94
(P36).- In your unit (or service, ward, department, primary care unit, etc.), good work is recognised and valued.	
(P37).- Your line manager has the necessary technical and management skills to perform the duties associated with his/her position.	
(P38).- You have a good professional relationship with your line manager.	
(P39).- You are satisfied with the opportunities to have to participate in the daily decision-making related to your work and working environment.	
(P40).- Your comments and suggestions to improve your unit (or service, ward, department, primary care unit, etc.) are listened to and taken into account.	
(P41).- You have sufficient opportunities to contribute to efforts to improve the operating (in terms of organisation, activities, etc.…) of your unit or area of activity.	
(P42).- In your unit, the work is well organised.	
(P43).- Collaborative processes are well defined between units that need to work together.	
(P44).- The objectives and action plans (describing the results expected from your work) established for your unit or area of influence are adequate.	
(P45).- You have clear work objectives.	
**Dimension 9. Policy and strategy**	0.92
(P46).- In your opinion, the management of your organisation (in terms of planning, objectives and organisation) is satisfactory.	
(P47).- You clearly understand the objectives, projects and results of your organisation.	
(P48).- You know enough about the mission and vision of your organisation.	
(P49).- In your organisation, good work is recognised.	
(P50).- The management in your organisation is open to suggestions and contributions from the staff.	
(P51).- In your organisation, it is really the case that there are equal opportunities for men and women for promotion and development.	
(P52).- Your organisation makes efforts to improve the way it works.	

The population covered by these surveys was the entire workforce (both permanent as well as temporary staff who had been working in the organisation for at least six months). Table 2 summarises data on the size and composition of the samples of employees who completed the questionnaire in each period.

**Table 2:** **Staff satisfaction survey sample size by assessment cycle and healthcare organisation**

Name of the healthcare organisation	2001-2003		2004-2006		2007-2008		2009-2010	
	Number of questionnaires	% staff	Number of questionnaires	% staff	Number of questionnaires	% staff	Number of questionnaires	% staff
Cruces Hospital	1,777	33.2	300	8.4	300	8.3	334	9
Basurto Hospital	1,050	43.6	200	8.2	352	14.3	340	13.5
Galdakao Hospital	392	31.8	562	43.9	328	25	325	24.1
Santiago Hospital	274	31.0	204	22.3	309	33.3	299	31
Txagorritxu Hospital	387	25.9	402	26.6	325	21.1	334	20.9
Donostia Hospital	1,622	47.9	1,071	30.9	373	10.7	361	10.1
San Eloy Hospital	n.a.^1^	55.9	186	39.2	245	51.9	238	48.3
Alto Deba Hospital	146	52.5	202	67.8	202	66.9	203	60.6
Bidasoa Hospital	186	56.4	229	67.2	217	63.8	203	53.8
Mendaro Hospital	n.a.^1^	55.3	203	54.9	204	54.8	228	55.1
Zumarraga Hospital	n.p.^2^	n.p.^2^	262	57.5	238	51.2	216	42.9
Gorliz Hospital	92	34.0	111	40.1	160	58.4	171	60.9
Santa Marina Hospital	120	50.8	n.p.^2^	n.p.^2^	198	82.5	165	64.5
Leza Hospital	40	39.6	49	45	101	96.2	62	54.4
Alava Primary care organization	366	66.0	250	44.7	402	70.9	383	61.8
Bilbao Primary care organization	369	45.8	350	43.6	357	44.5	469	55.6
Ezkerraldea-Enkarterri Primary care organization	447	61.3	401	56.8	421	59.6	375	53.1
Interior Primary care organization	388	54.5	336	48	350	49.7	370	48.2
Uribe Primary care organization	211	54.2	200	50.9	256	64.3	264	57.3
Gipuzkoa Este Primary care organization	460	56.6	490	60.8	411	50.7	515	56.5
Gipuzkoa Oeste Primary care organization	321	43.2	n.a.^1^	44.6	413	58.6	339	45.8
Bermeo Psychiatric hospital	89	41.9	97	44.5	165	77.5	435^3^	49.7^3^
Zaldibar Psychiatric hospital	133	64.7	58	27.6	131	63.9		
Zamudio Psychiatric hospital	80	33.3	200	85.5	156	68.1		
Bizkaia Mental health outpatient service	147	61.7	159	65.2	204	84.3		
Alava Psychiatric hospital and mental health outpatient service	161	44.6	n.a.^1^	57.6	206	56.9	216	58.7
Gipuzkoa Mental health outpatient service	78	72.6	n.a.^1^	70.4	116	84.1	95	67.4
Basque transfusion and human tissue Centre	41	69.2	62	98.4	99	n.a.^1^	44	61.1
Emergency services provider	91	58.9	103	56.3	228	n.a.^1^	107	48.4
Central office	264	62.3	145	41.4	184	53.8	161	39.7

^1^n.a.: not available; ^2^n.p.: did not participate in survey; ^3^In the cycle 2009–2010, Bermeo, Zaldibar and Zamudio hospitals and Bizkaia mental health outpatient were merged into a single organization (Bizkaia Mental Health Network).

For this study, data were collected for 30 healthcare organisations including acute, psychiatric, and medium- and long-stay hospitals, as well as providers of outpatient mental health services and primary care. The statistical analysis was carried out independently for each of the four assessment cycles studied and, within each cycle, data were grouped into categories to allow two separate comparisons: i) organisations that had received some type of Q Award (regardless of whether it was Silver or Gold) vs. those without this type of external recognition; and ii) organisations with a Gold Q vs. those with no awards. In a given assessment cycle, organisations were considered to have received external recognition if they had been given a Q Award during or at any time prior to the corresponding cycle.

Table 3 shows the sizes of the resulting groups for each assessment cycle. As can be seen, the sample size is relatively small in some cases. For instance, by the end of the first cycle, only one organisation had obtained a Gold Q Award. As the statistical techniques used cannot be applied to a single observation, for this cycle it was not possible to make the comparison between recipients of Gold Q Awards and organisations without this type of external recognition. Further, in two groups there are just four organisations (the group with some type of Q Award in 2001–2003, and that with Gold Q Awards in 2004–2006); for these cases, the results should be interpreted with caution.

**Table 3:** **Number of healthcare organisations in each study group by assessment cycle**

	Assessment cycle			
	2001-2003	2004-2006	2007-2008	2009-2010
**Number of healthcare organisations with no Q Certification**	**25**	**14**	**11**	**11**
**Number of healthcare organisations with Q Certification**	**4**	**15**	**19**	**19**
Recipients of a Silver Q	3	11	13	12
Recipients of a Gold Q	1	4	6	7
**Total number of healthcare organisations per cycle**	**29**	**29**	**30**	**30**

Given that the objective of this study was to assess whether organisations with Q Awards were rated more highly than those without this type of external recognition, the null hypothesis of equality of the means was tested, with a one-tailed independent samples *t*-test. Prior to this test, however, the Shapiro-Wilk test was conducted to check whether the data was normally distributed. In cases where the normality assumption was violated, non-parametric tests such as the Kruskal-Wallis test were used instead.

This study is framed within a research project that was presented and approved in a commissioned research call granted by the Basque Office for Health Technology Assessment (Osteba) an agency of the Basque Government’s Department of Health. During the project approval process, it was externally reviewed and evaluated and it was decided that it is not necessary to have an Ethics Committee approval in order to ensure compliance with existing conventions and standards in research.

The results of the questionnaires are based on Osakidetza’s (Basque Health Service) professionals responses and were provided by the Quality and Human Resources Department of Osakidetza. Involved professionals participation was voluntary and anonymous, and obtained data was treated as absolutely confidential and anonymous, according to Spanish data privacy law.

## Results

The results of the comparisons between organisations which had and had not received Q Awards and between recipients of Gold Q Awards and those without this type of external recognition are summarised in Tables 4 and 5. Each cell contains, for each assessment cycle and dimension analysed, the differences observed in the mean scores (on a scale from 0 to 10) for those which had and had not received the awards and the standard deviation (in brackets).

**Table 4:** **Differences in mean results (** ***t*** **-test) on staff satisfaction questionnaire (0–10 scale) between the group of organisations with Q certification (Silver or Gold) and those without certification, per cycle and dimension**

Dimensions	Differences in mean value on staff satisfaction			
	(standard deviation)			
	2001-2003	2004-2006	2007-2008	2009-2010
**Internal communication**	0.39 (0.35)	**0.74 (0.18)**	**0.40 (0.16)**	**0.27 (0.14)**
**Training and professional development**	0.35*	**0.63 (0.12)**	**0.33 (0.17)**	**0.28 (0.15)**
**Policy and strategy**	0.43 (0.44)	**0.68 (0.19)**	0.31 (0.20)	**0.31 (0.17)**
**Leadership and management style in the unit/service**	0.28 (0.27)	**0.71 (0.13)**	0.28 (0.16)	0.18 (0.12)
**Technical and material resources**	0.60*	**0.39 (0.18)**	0.14 (0.18)	0.34*
**Health and safety at work**	0.32 (0.29)	**0.61 (0.20)**	0.14 (0.17)	0.03 (0.15)
**Working conditions**	0.05 (0.34)	0.29 (0.18)	0.25 (0.17)	0.19 (0.13)
**Working environment**	0.05 (0.21)	0.27 (0.18)	0.20 (0.15)	0.14 (0.09)
**Pay**	-0.09 (0.29)	0.45*	0.25 (0.18)	0.12 (0.13)

*For this dimension and cycle, the non parametric Kruskal – Wallis test was used instead of the *t*-test, because the normality assumption was violated.

Mean values in bold are those with p < 0.05.

**Table 5:** **Differences in mean results (t- test) on staff satisfaction questionnaire (0–10 scale) between the group of organisations with Gold Q Award and those with no Q certification, per cycle and dimension**

Dimensions	Differences in mean value on staff satisfaction		
	*(standard deviation)		
	2004-2006	2007-2008	2009-2010
**Internal communication**	**0.75 (0.25)**	**0.54 (0.20)**	**0.37 (0.15)**
**Training and professional development**	**0.72 (0.12)**	**0.38 (0.18)**	**0.40 (0.12)**
**Policy and strategy**	**0.60 (0.28)**	**0.49 (0.20)**	**0.40 (0.18)**
**Leadership and management style in the unit/service**	**0.63 (0.18)**	**0.43 (0.21)**	**0.24 (0.13)**
**Technical and material resources**	0.30 (0.26)	0.07 (0.23)	**0.44 (0.21)**
**Health and safety at work**	0.29 (0.23)	0.24 (0.20)	0.22 (0.18)
**Working conditions**	0.09 (0.24)	**0.32 (0.17)**	0.24 (0.14)
**Working environment**	0.21 (0.26)	0.18 (0.23)	0.16 (0.13)
**Pay**	0.04 (0.31)	0.10 (0.23)	0.13 (0.14)

*Mean values in bold are those with p < 0.05.

Regarding the level of significance of these differences, the cells with bold numbers correspond to the cases when the one-sided *t*-test produced a p-value of less than 0.05. This means that for these dimensions there was sufficient evidence in these cycles to state, with a confidence of 95%, that organisations which do have external recognition were rated more highly by their employees than those which do not. Four cells in Table 4 are marked with an asterisk indicating that, as the normality assumption was violated, the value in the cell corresponds to the result of the non-parametric Kruskal-Wallis test, rather than the *t*-test. Accordingly, the standard deviation is not given for these cases.

### Comparison between organisations with some type of Q Award vs. those with none

First, we present the comparison between recipients of some type of Q Award (Gold or Silver) and those without any Q Awards (Table 4). In the first assessment cycle (2001–2003), statistically significant results were only found for one dimension. The group of organisations that had received Q Awards was small, however, and we should, therefore, be cautious in the interpretation of these results.

**Figure 1:**
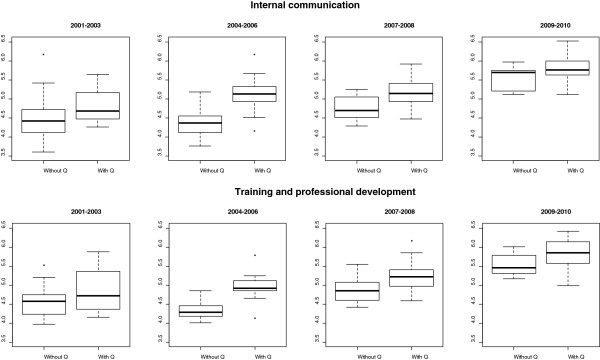
**Boxplots comparing the groups for the two variables that showed largest differences throughout the entire period.**

In the other periods, ratings were consistently higher in the group with, compared to that without Q Awards, in two dimensions (p <0.05): Training and professional development, and Internal communication (Figure 1). In addition, although with a lower level of significance (0.05 < p < 0.10), organisations with Q Awards performed better in the areas related to Leadership and management style and Policy and strategy. A similar trend was observed in indicators concerning Working conditions and Working environment. In most of the periods, however, it was not possible to reject the null hypothesis that the means were equal for the areas of Health and safety at work and Pay.

### Comparison between organisations with Gold Q Awards vs. those with no awards

The comparison between recipients of Q Gold Award and those without any Q Awards is presented in Table 5. In the first period, it was not possible to compare results for recipients of Gold Q Awards with those that had received no awards as the sample size was not sufficiently large. In the rest of the periods, consistent with the fact that there were greater differences in terms of degree of implementation of the EFQM model between these groups, the pattern was much more clear for the four dimensions identified in the first analysis, namely those related to Training and professional development, Internal communication, Leadership style and Policy and strategy. These differences between organisations with Gold Q Awards and those with no awards were highly significant for all the three periods considered. On the other hand, for the dimension related to Working conditions, the differences were only significant in two periods and less strongly so. As regards Health and safety at work and Pay, no significant differences were found between the groups in any of the assessment cycles. There were also no significant differences for Working environment. For Technical and material resources there was only a significant difference in the last assessment cycle.

## Discussion

The results in this study indicate that the satisfaction of health service employees in organisations that have made the most progress towards the implementation of the EFQM model (as reflected in the possession of a Gold Q Award) was significantly greater in several areas, namely training, internal communication, leadership style and strategy/policy, than among those working for organisations that have not gone so far in the implementation of the model (without any Q award). These differences were still significant in the training and communication factors when comparing recipients of any type of Q Award with those who have yet to receive this type of external recognition.

Comparing the differences in the two analysis (with vs. without Q Awards, and with a Gold Q vs. without any of this type of award), in the periods 2007–2008 and 2009–2010, we found that the magnitude of the differences between organisations with a Gold Q Award (those that had made more progress in terms of the EFQM) and those without any awards is greater than between organisations with and without any type of Q Award, across all of the dimensions with highly significant differences (p <0.05). It seems therefore that the differences in term of satisfaction increase with progressive deployment of the model (being the greater among employees of organisations with Gold than Silver Q Awards).

This study contributes to the evidence showing the long-term impact of a complex organisational intervention such as the adoption of the EFQM model in a series of healthcare organisations within a single public entity, Osakidetza. In fact, there is little data in the literature on the implementation of the EFQM model in healthcare settings and results have not been conclusive [19]. Further, while other authors have reached similar conclusions, none of the previously reported studies have covered as broad a period of time or as many organisations (in terms of diversity and number) as the present one. For instance, the positive effect of employees’ satisfaction on organisational improvement has been demonstrated by some authors [23], while it is also recognised that it is difficult to quantify and assess its impact on outcome indicators [24] or on the general performance of the organisation.

This study has various limitations. On the one hand, although external recognition as a criterion for assessing progress towards establishment of the EFQM model has been used by other authors [16, 18], this approach may be questionable as an organisation could decide not to apply for the awards, despite having adopted the model. In the case of Osakidetza, this circumstance is improbable given that the corporate strategy of the health service (as set out in the Strategic Plans [12–14]) explicitly encourages member organisations to obtain these types of external recognition.

On the other hand, a wide range of factors may affect employee satisfaction, and hence attributing any observed effect to the deployment of the EFQM model is questionable. Nevertheless, the pattern of differences observed seems to support the hypothesis of the management model having an impact on the results, namely because the areas in which significant differences were found corresponded to those in which there is the greatest margin for independent management at the level of the separate healthcare organisations (training, internal communication, leadership style, etc.), while those in which differences were not significant were related to common corporative elements, more homogeneous across all the organisations in Osakidetza (such as health and safety at work and pay).

Another factor that could have affected the differences between organisations to some extent is that all the healthcare organisations within Osakidetza have been exposed to the EFQM model and that all of them have adopted it to a greater or lesser extent. Despite this, we found significant differences for organisations judged to have made the most progress, by means of a greater degree of implementation of the model and seeking (and being granted) external recognition, which were rated more highly in terms of employee satisfaction than those that have made less progress.

Finally, in the comparison between organisations granted Gold Q Awards vs. those without any Q Awards, the effect of the model was seen to be maintained over time, and some positive effects even appeared in the last assessment period, namely in relation to satisfaction with technical and material resources. In the comparisons between organisations with any Q Awards versus those with no Q Awards, however, there seems to be a reduction in the effect with time. Although the results of the surveys are not fully comparable between assessment cycles, such an effect could be attributable to an organisation making less effort once it had obtained a Silver Q award. In fact, a qualitative analysis associated with this study also indicated that organisations to some extent overstrained to obtain the Silver Q award and after that tended to relax, while the Gold Q Award takes longer to be awarded and is granted in a more mature setting and, therefore, the conditions are more stable. This apparent reduction in the differences (to be interpreted with caution given the limitations in terms of comparability between the surveys carried out in the different periods), could also be due to a narrowing of differences over time between organisations with a lower level of external recognition (Silver Q award) and without external award, as they have all been exposed to quality promotion policies from the corporate level.

## Conclusions

The progress made by Osakidetza healthcare organisations in the implementation of the EFQM model is associated with a greater level of employee satisfaction in fields that can be managed independently by each organisation, while no significant differences were observed in those aspects that are more homogeneously and centrally managed across the regional health service and in which each organisation has less scope for self-management.

This study also shows that the greater the progress in the implementation of the model (as in the case of the organisations with Gold Q Awards compared to those with Silver Q Awards), the greater the differences in terms of staff satisfaction with the less advanced organisations (those without any external recognition) and in a broader range of dimensions.

## References

[1] Euskalit: *Fundación Vasca para la Excelencia*. http://www.euskalit.net Accessed [30 May 2012]

[2] European Foundation for Quality Management (2003). EFQM. Modelo EFQM de Excelencia.

[3] Arcelay A, Hernández L, Inclán G, Bacigalupe M, Letona J, Linares R (1998). Proceso de autoevaluación de los centros sanitarios de Osakidetza mediante el modelo europeo de gestión de la calidad total. Revista Calidad Asistencial.

[4] Arcelay A, Sánchez E, Hernández L, Inclán G, Bacigalupe M, Letona J, González RM, Martínez-Conde AE (1999). Self-assessment of all the health centres of a public health service through the European Model of Total Quality Management. Int J Health Care Q Assurance.

[5] Sánchez E, Darpón J, Villar F, Letona J, Martínez-Conde A, González LR (2000). De la gestión de la calidad hacia la excelencia en la gestión a través del modelo de autoevaluación de la European Foundation for Quality Management (EFQM) en una red pública de centros sanitarios. Revista Calidad Asistencial.

[6] Sánchez E, Darpón J, Garay JI, Letona J, González R, Pérez MJ (2004). Política de calidad en Osakidetza-Servicio vasco de salud. Revista Calidad Asistencial.

[7] Muñoz J, González R, Letona J, Sánchez E, García M, Ballesteros C (2005). Herramienta de Autoevaluación Rápida Para Organizaciones Sanitarias Según el Modelo de Excelencia EFQM.

[8] Aramburu I, Vázquez S, Letona J (2005). Guía Para la Auto-Implantación de la Metodología 5S en Organizaciones Sanitarias.

[9] Osakidetza (1990). Guía Para la Gestión de Procesos.

[10] Pérez MJ, Letona J, Sánchez E, Audícana A, Gutiérrez F, San MA (2003). Implantación de la gestión por procesos en una corporación sanitaria a través de la certificación según la norma ISO 9001:2000 [abstract]. Revista Calidad Asistencial.

[11] Audicana A, Letona J, Pérez MJ, San Martín A, Sánchez E (2004). Guía de Gestión por Procesos e ISO 9001:2000 en las Organizaciones Sanitarias.

[12] Osakidetza (1998). Plan Estratégico 1998–2002.

[13] Osakidetza (2003). Plan Estratégico 2003–2007.

[14] Osakidetza (2008). Plan Estratégico 2008–2012.

[15] Osakidetza (2001). Manual de Evaluación y Mejora de la Satisfacción de las Personas en las Organizaciones de Servicios.

[16] Hendricks KB, Singhal VR (1997). Does implementing an effective TQM program actually improve operating performance? Empirical evidence from firms that have won quality awards. Manag Sci.

[17] Santos-Vijande ML, Alvarez-Gonzalez LI (2007). TQM and firms performance: An EFQM excellence model research based survey. Int J Bus Sci Appl Manage.

[18] Boulter L, Bendell T, Abas H, Dahlgaard J, Singhal V (2005). Report on EFQM and BQF Funded Study into the Impact of the Effective Implementation of Organisational Excellence Strategies on Key Performance Results.

[19] Minkman M, Ahaus K, Huijsman R (2007). Performance improvement based on integrated quality management models: what evidence do we have? A systematic literature review. Int J Qual Health Care.

[20] Sánchez E, Letona J, González R, García M, Darpón J, Garay JI (2006). A descriptive study of the implementation of the EFQM excellence model and underlying tools in the Basque Health Service. Int J Qual Health Care.

[21] Goldstein SM, Schweikhart SB (2002). Empirical support for the Baldrige Award framework in U.S. hospitals. Health Care Manag Rev.

[22] Shortell SM, O'Brien JL, Carman JM, Foster RW, Hughes EF, Boerstler H, O'Connor EJ (1995). Assessing the impact of continuous quality improvement/total quality management: concept versus implementation. Health Serv Res.

[23] Lin MK, Marsteller JA, Shortell SM, Mendel P, Pearson M, Rosen M, Wu SY (2005). Motivation to change chronic illness care: results from a national evaluation of quality improvement collaboratives. Health Care Manag Rev.

[24] Shortell SM, Jones RH, Rademaker AW, Gillies RR, Dranove DS, Hughes EF, Budetti PP, Reynolds KS, Huang CF (2000). Assessing the impact of total quality management and organizational culture on multiple outcomes of care for coronary artery bypass graft surgery patients. Med Care.

[CR25] The pre-publication history for this paper can be accessed here:http://www.biomedcentral.com/1472-6963/14/468/prepub

